# Mu opioid receptor mRNA overexpression predicts poor prognosis among 18 common solid cancers: A pan-cancer analysis

**DOI:** 10.3389/fonc.2023.1134744

**Published:** 2023-03-30

**Authors:** Wei Sun, Shaohui Zhuang, Minghua Cheng, Zeting Qiu

**Affiliations:** Department of Anesthesiology, The First Affiliated Hospital of Shantou University Medical College, Shantou, China

**Keywords:** Mu opioid receptor, pan-cancer analysis, prognostic features, solid cancer, opioid

## Abstract

**Background:**

Opioids are widely used for patients with solid tumors during surgery and for cancer pain relief. We conducted a pan-cancer genomic analysis to investigate the prognostic features of Mu opioid receptor (*MOR*) mRNA expression across 18 primary solid cancers.

**Methods:**

All the data of cancer with *MOR* mRNA were retrieved from cBioPortal for Cancer Genomics. Logistic regression was used to determine the associations between *MOR* mRNA expression and clinicopathological features. Log-rank test and Cox regression was used for survival analysis. Subgroup analysis and propensity score matching were also carried out.

**Results:**

7,274 patients, including 1,112 patients with positive *MOR* mRNA expression, were included for data analyses. Positive *MOR* mRNA expression was associated with more advanced stage of T (adjusted Odds ratio [OR], 1.176; 95% confidence interval [CI], 1.022-1.354; *P*=0.024), M (adjusted OR, 1.548; 95% CI, 1.095-2.189; *P*=0.013) except N (adjusted OR, 1.145; 95% CI, 0.975-1.346; *P*=0.101), and worse prognosis for overall survival (Hazard ratio [HR] 1.347, 95% CI 1.200-1.512, *P*<0.001), progression-free survival (HR 1.359, 95% CI 1.220-1.513, *P*<0.001), disease-free survival (HR 1.269, 95% CI 1.016-1.585, *P*<0.001) and disease-specific survival (HR 1.474, 95% CI 1.284-1.693, *P*<0.001). Patients with positive *MOR* mRNA expression tended to be classified as tumor microenvironment immune types II, representing low PD-L1 and low CD8A expression.

**Conclusion:**

*MOR* mRNA overexpression is associated with poor prognosis and poor response to PD-L1 therapy.

## 1 Introduction

Cancer is still a common cause of death globally although study into molecular mechanisms of cancer cell biology and treatments including immunotherapy are well advancing. However, surgery is still the frontline treatment of solid tumors (1). Sadly, cancer reoccurrence followings surgery is the main cause of death. This may be due to many factors including the malignant nature of disease, surgical stress and inflammatory responses. But beyond these, anesthetics/techniques used during the perioperative period may also contribute to cancer reoccurrence and patients’ death (2–4). In addition, opioids are widely used for cancer patients during surgery and beyond such as intraoperative anesthesia, postoperative analgesia, and advanced cancer pain relief. Opioids work through acting on opioid receptors expressed in the central and peripheral neurons, and then reducing pain transduction to the central nervous system (5). There are three sub-types of opioid receptors, namely Mu opioid receptors (MOR), Delta opioid receptors and Kappa opioid receptors (6). MOR is the primary receptor for endogenous opioids like endorphin and enkephalins, as well as exogenous opioids like morphine and fentanyl. It is a prototypical G protein-coupled receptor that plays an important role in regulating pain, reward, and addictive behaviors (7).

Retrospective studies suggested that increased opioid use during cancer surgery may be related to cancer recurrence (8). Subsequent studies found that high MOR expression indicated poor prognosis in a variety of cancers including lung cancer, hepatocellular carcinoma and esophageal carcinoma (9–11). Furthermore, *in vivo* and *in vitro* experimental data also suggested that MOR was involved in tumor proliferation and metastasis (11). MOR may also regulate the immune system through mediating immune suppression (12).

The relationship between *MOR* and various solid cancers on long term surgical outcomes is limited. In this study, *MOR* mRNA expression across 18 solid cancers was analyzed based on the integrative pan-cancer TCGA database (13). The significant role of *MOR* mRNA expression in clinicopathological characteristics and prognosis were reported together along with its immunogenic features in the current study.

## 2 Materials and methods

### 2.1 Data sources

The public and de-identified data of primary solid tumors from TCGA database by cBioPortal for Cancer Genomics (https://www.cbioportal.org, accessed in March 25, 2020), including genomic, demographic, clinicopathological and prognostic data were retrieved (14). The genomic data consisted of mRNA-Seq expression data, which was generated using Illumina HiSeq V2 platform. The mRNA-Seq data were processed and normalized using RNA-Seq by expectation maximization, and transformed into log 2 values for analysis (15). Our pan-cancer analysis only focused on Mu Opioid Receptor mRNA expression in 18 common primary solid tumors (**Table S1**) and any patients whose *MOR* mRNA expression was not available were excluded.

### 2.2 Variable selection

The *MOR* mRNA-Seq expression data (symbol: OPRM1; gene ID: 4988) of 18 common solid cancer types were retrieved from the TCGA database. Initially, we divided all patients into positive versus negative subgroups by median *MOR* mRNA expression values of each cancer type (16). Then we found *MOR* mRNA expression was at a low level, and most median values were zero. Finally, we defined patients with zero *MOR* expression as negative. Demographic data included age, gender and race. Age was classified as young (under 60 years old), old (over 60 years old) and unknown subgroups (the median of age was 60 years old). Gender was classified as male, female and unknown. Race was classified as Caucasian, African and others. Clinicopathological data were the American Joint Committee on Cancer (AJCC) Tumor-node-metastasis (TNM) stages and histological grade. AJCC pathologic TNM stage was classified as stage I, stage II, stage III, stage IV and others. AJCC pathologic T stage was classified as T1, T2, T3, T4 and others. N stage was classified as N0, N1, N2, N3 and others. M stage was classified as M0, M1 and others. Histological grade was classified as low grade, high grade and others. In this study, when investigating the prognostic features of *MOR*, we focused on overall survival (OS), progression-free survival (PFS), disease-free survival (DFS) and disease-specific survival (DSS).

We also analyzed the association between *MOR* mRNA expression and tumor microenvironmental immune types (TMIT) and assessed the immunogenic features of the *MOR*. According to previous studies (17), the TMIT classification was divided into high or low expressions based on the expression of PD-L1 and CD8A. The type I represents high PD-L1 and high CD8A expression, type II with low PD-L1 and low CD8A expression, type III as high PD-L1 and low CD8A expression, and type IV of low PD-L1 and high CD8A expression. To explain, TMIT type II implies decreased infiltration of CD8+ T cells in the tumor microenvironment and decreased expression of PD-L1 in cancer cells, which represents a poor response to PD-L1 therapy.

### 2.3 Statistical analysis

Continuous variables with normal distribution were described as mean and standard deviation. Continuous variables with skewed distribution were described as median, first quartile and third quartile. Categorical variables were described as frequency and percentage. Pearson’s Chi-squared test or Fisher’s exact test was used to detect the statistical significance for categorical variables of demographic and clinicopathological features between *MOR* subgroups, as well as the association between TMIT and *MOR* mRNA expression. Independent Student’s t-test was used to detect a statistical significance for continuous variables with normal distribution and homogeneity of variances between *MOR* subgroups; Otherwise, Kruskal-Wallis test was used. Binomial logistic regression models were used to detect associations between *MOR* mRNA expression and binary clinicopathological features. Multinomial logistic regression models were used to detect the associations between *MOR* mRNA expression and polyfactorial clinicopathological features. The greater odds ratio (OR) value indicates the more advanced stage and grade of cancer. The log-rank test and Kaplan-Meier estimator were used to screen significant prognostic factors that were associated with survival outcomes. Glioblastoma multiforme (GBM) and skin cutaneous melanoma (SKCM) were excluded from the disease-free survival analysis, because no disease-free survival data was available for GBM and SKCM. After adjustment of Cox regression model, the hazard ratio (HR) for each prognostic factor was calculated. The greater HR value suggested the greater risk of death. To eliminate potential disequilibrium caused by confounding factors, subgroup analysis and propensity score matching (PSM) were done. All statistical analysis was done by R statistical software (version 3.6.2, released on February 29, 2020). A Two-sided *P* value < 0.05 was considered to be of statistical significance.

## 3 Results

### 3.1 Patients’ demographics across 18 solid cancer types

There were 7,274 patients with 18 common solid cancer types included into this study; Of those, 6126 patients were with negative *MOR* mRNA expression and 1,112 patients with positive *MOR* mRNA expression (**Table 1**). There were 3,630 young patients with age up to 60 years old, 3,612 male patients, and 5, 222 Caucasian patients (**Table 1**). The majority of patients belonged to stage I (1755), N0 stage (3247) and M0 stage (3872) (**Table 1**). Generally, the positive rates of *MOR* mRNA expression varied from 2.7% to 50.2% across different cancer types (**Table S1**). The clinicopathological data were missing in some cancer types, and these cancer types were excluded in subsequent analysis. Compared with normal tissues, *MOR* mRNA was generally expressed in solid cancers, but the mRNA expression level was relatively low (**Figure S1**). The colon adenocarcinoma (COAD) and rectal adenocarcinoma (READ) had similar embryological and histological features and they were classified as one group (colon and rectal adenocarcinoma, COREAD) in the following analysis.

**Table 1:** Baseline characteristics of the included patients in the TCGA database.

Characteristic	Total	*MOR* (-)	*MOR* (+)
	N = 7274	N = 6162	N = 1112
Age	59.0 ± 14.2	59.3 ± 14.0	57.4 ± 15.2
Age group			
Young	3630	3059 (49.6)	571 (51.3)
Old	3542	3024 (49.1)	518 (46.6)
Gender			
Female	3604	3114 (50.5)	490 (44.1)
Male	3612	3003 (48.7)	609 (54.8)
Race			
Caucasian	5222	4376 (71.0)	846 (76.1)
African	686	581 (9.4)	105 (9.4)
AJCC stage			
Stage I	1755	1556 (25.3)	199 (17.9)
Stage II	1710	1492 (24.2)	218 (19.6)
Stage III	1281	1146 (18.6)	135 (12.1)
Stage IV	689	571 (9.3)	118 (10.6)
Grade			
Low Grade	1392	1073 (17.4)	319 (28.7)
High Grade	1490	1158 (18.8)	332 (29.9)
AJCC-T			
T1	1556	1404 (22.8)	152 (13.7)
T2	2205	1921 (31.2)	284 (25.5)
T3	1817	1599 (25.9)	218 (19.6)
T4	554	463 (7.5)	91 (8.2)
AJCC-N			
N0	3247	2860 (46.4)	387 (34.8)
N1	1380	1203 (19.5)	177 (15.9)
N2	589	497 (8.1)	92 (8.3)
N3	154	144 (2.3)	10 (0.9)
AJCC-M			
M0	3872	3435 (55.7)	437 (39.3)
M1	251	208 (3.4)	43 (3.9)

MOR (+) represented positive MOR mRNA expression; MOR (-) represented negative MOR mRNA expression; Young, under 60 years old; Old, over 60 years old; All the variables were described as frequencies and percentages, except for age described as mean and standard deviation.

TCGA, the Cancer Genome Atlas; MOR, mu opioid receptor; AJCC, the American Joint Committee on Cancer; TNM, Tumor node metastasis.

### 3.2 Clinicopathological features of Mu opioid receptor across cancer types

**Figure 1:**
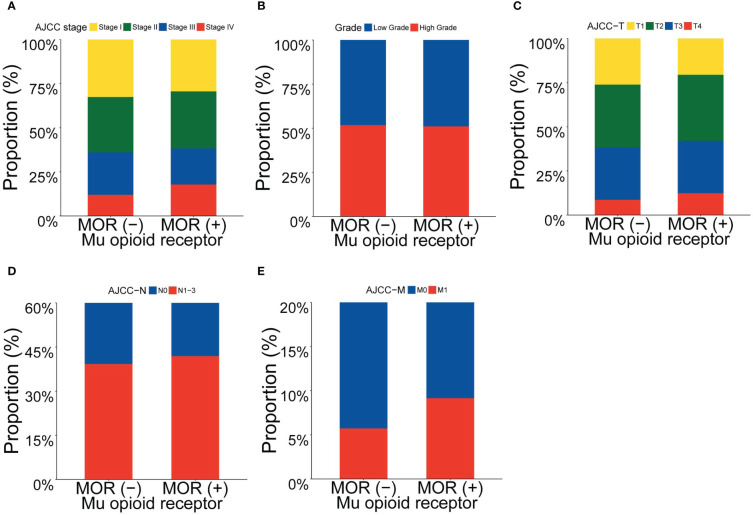
Proportion of clinicopathological features according to *MOR* mRNA expression. Footnotes: Proportion of **(A)** AJCC stage, **(B)** grade, **(C)** AJCC T stage, **(D)** AJCC N stage and **(E)** AJCC M stage.

We explored the association between clinicopathological features (including AJCC TNM stage, histological grade, T stage, N stage and M stage) and mRNA expression of *MOR* across 18 major solid cancer types. Patients with positive *MOR* mRNA expression tended to have more advanced AJCC TNM stage, T stage, N stage and M stage when compared with patients with negative *MOR* mRNA expression (**Figure 1**). There was no visible relationship between *MOR* expression and histological grade. In the following analysis, we combined N1, N2 and N3 together.

After adjustment of multivariate logistic regression analysis, as shown in **Table 2**, we found that the association between T stage and *MOR* mRNA expression remained statistically significant (adjusted OR, 1.176; 95% confidence interval [CI], 1.022-1.354; *P*=0.024), as well as the association between M stage and *MOR* mRNA expression (adjusted OR, 1.548; 95% CI, 1.095-2.189; *P*=0.013), but association between N stage and *MOR* was not significant statistically (adjusted OR, 1.145; 95% CI, 0.975-1.346; *P*=0.101). In the sensitivity analysis, we removed one cancer type at each time and then re-analyze the association between clinicopathological features and *MOR* mRNA expression. We found that the association between T stage, M stage and *MOR* mRNA expression was stable (**Figure S2**). Subgroup analysis showed that there was an association trend between clinicopathological features and *MOR* mRNA expression (most adjusted OR>1.0), but the statistical difference is not significant (most *P*>0.05), (**Figure S3**).

**Table 2:** The association between *MOR* mRNA expression and clinicopathological features overall.

Response variable	Adjusted OR	95% CI	*P*-value
AJCC TNM stage	1.135	0.978-1.318	0.096
Grade	1.011	0.846-1.207	0.908
AJCC-T	1.176	1.022-1.354	0.024
AJCC-N	1.145	0.975-1.346	0.101
AJCC-M	1.548	1.095-2.189	0.013

MOR (+) represented positive MOR mRNA expression; MOR (-) represented negative MOR mRNA expression; Young, under 60 years old; Old, over 60 years old; All the variables were described as frequencies and percentages, except for age described as mean and standard deviation.

TCGA, the Cancer Genome Atlas; MOR, mu opioid receptor; AJCC, the American Joint Committee on Cancer; TNM, Tumor node metastasis.

### 3.3 Mu opioid receptor mRNA as a potential prognostic biomarker across cancer types

There were 43 patients (0.6%) with missing OS data, 43 patients (0.6%) with missing PFS data, 3529 patients (48.5%) with missing DFS data, and 275 patients (3.8%) with missing DSS data. These patients were excluded from the corresponding survival analysis. The median follow-up periods were 23.87 months (13.12-46.45 months) for OS, 19.79 months (10.00-39.45 months) for PFS, 23.74 months (13.71-43.73 months) for DFS, and 23.87 months (13.25-46.32 months) for DSS.

**Figure 2:**
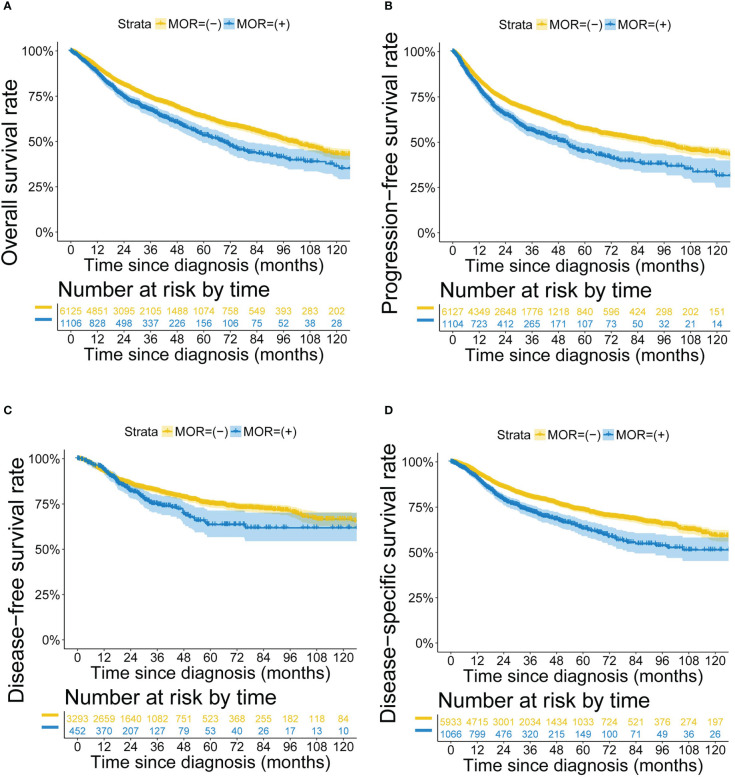
Kaplan-Meier survival curves according to *MOR* mRNA expression. Footnotes: Kaplan-Meier curves for **(A)** OS, **(B)** PFS, **(C)** DFS and **(D)** DSS.

The prognosis of patients with positive *MOR* mRNA expression was worse than patients with negative *MOR* expression (*P*<0.001 for OS, PFS, DFS and DSS) (**Figure 2**). The poor prognosis of positive *MOR* mRNA expression was observed in most cancer types (**Figures S4-S7**). Multivariate Cox regression models identified positive *MOR* mRNA expression as a significant prognostic factor in all cancer types (HR 1.347, 95% CI 1.200-1.512, *P*<0.001 for OS; HR 1.359, 95% CI 1.220-1.513, *P*<0.001 for PFS; HR 1.269, 95% CI 1.016-1.585, *P*<0.001 for DFS; HR 1.474, 95% CI 1.284-1.693, *P*<0.001 for DSS) (**Figure S8**). Sensitivity analysis confirmed the prognostic significance of *MOR* mRNA expression (**Figure S8**). After removing liver hepatocellular carcinoma (LIHC) from the study cohort, the effect of positive *MOR* mRNA expression on DFS turned more remarkable (HR 1.539, 95% CI 1.218−1.944, *P*<0.001) (**Figure S8**). We applied PSM to generate balanced data from the raw data, in which the demographics differences were eliminated between the two groups (**Table S2**). The adverse effect of positive *MOR* expression on prognosis still existed (*P*<0.001 for OS, PFS, DFS and DSS) with the balanced data (**Figure S9**).

### 3.4 Association between Mu opioid receptor mRNA and tumor microenvironment immune types

We grouped all the patients into four TMIT groups according to the expression levels of CD8A and PD-L1. Among the included patients, 35.0% were classified as TMIT I, with high PD-L1 expression and high CD8A expression. High PD-L1 expression indicated a favorable response to the PD-L1 therapies. High CD8A expression represented a high proportion of CD8+ CTLs in the tumor microenvironment. The proportions of TMIT II (low PD-L1/low CD8A), III (high PD-L1/low CD8A), and IV (low PD-L1/high CD8A) were 35.0%, 15.0% and 15.0%, respectively (**Table S3**).

**Figure 3:**
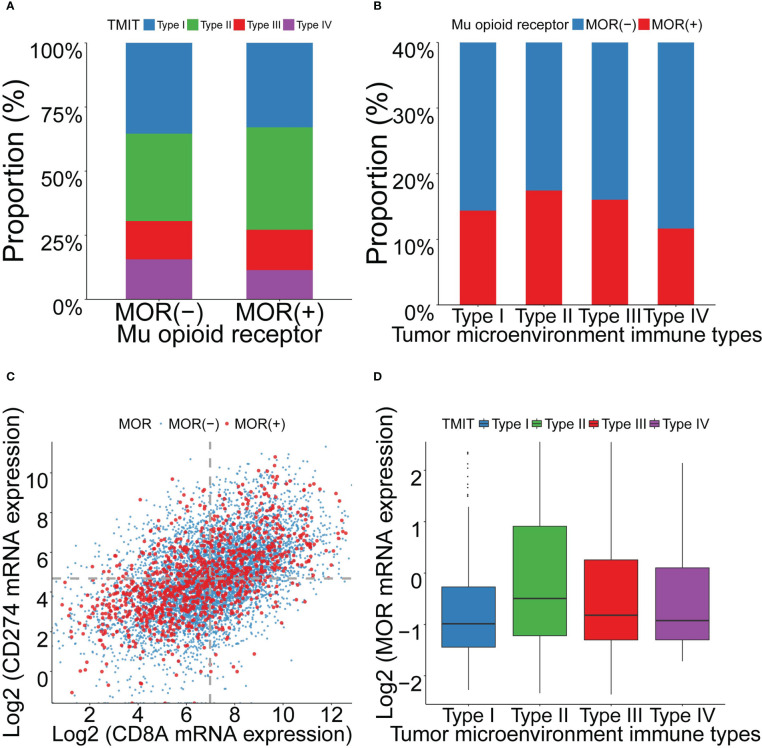
Association between *MOR* mRNA expression and TMITs, as well as correlation between PD-L1 and CD8A expression across 18 cancer types. Footnotes: **(A)** Distribution of TMITs according to *MOR* expression. **(B)** Distribution of *MOR* expression according to TMITs. **(C)** Correlation between mRNA expression level of PD-L1 and CD8A. **(D)** The mRNA expression level of *MOR* according to TMITs.

We analyzed the relationship between *MOR* mRNA expression and TMIT. As patients with positive *MOR* mRNA expression were likely to be classified as TMIT II, while patients with negative *MOR* expression were likely to be classified as TMIT I (**Figure 3A**). Patients of TMIT II had higher *MOR* mRNA expression, while patients of TMIT I had lower *MOR* expression (**Figures 3B, D** and **Figure S10**). This pattern remained in breast invasive carcinoma (BLCA), cervical squamous cell carcinoma (CESC), head and neck squamous cell carcinoma (HNSC), lung squamous cell carcinoma (LUSC). The expression levels of PD-L1 and CD8A were positively correlated overall and in most cancer types (**Figure 3C** and **Figure S11**).

### 3.5 Summary of clinical implications of Mu opioid receptor

**Figure 4:**
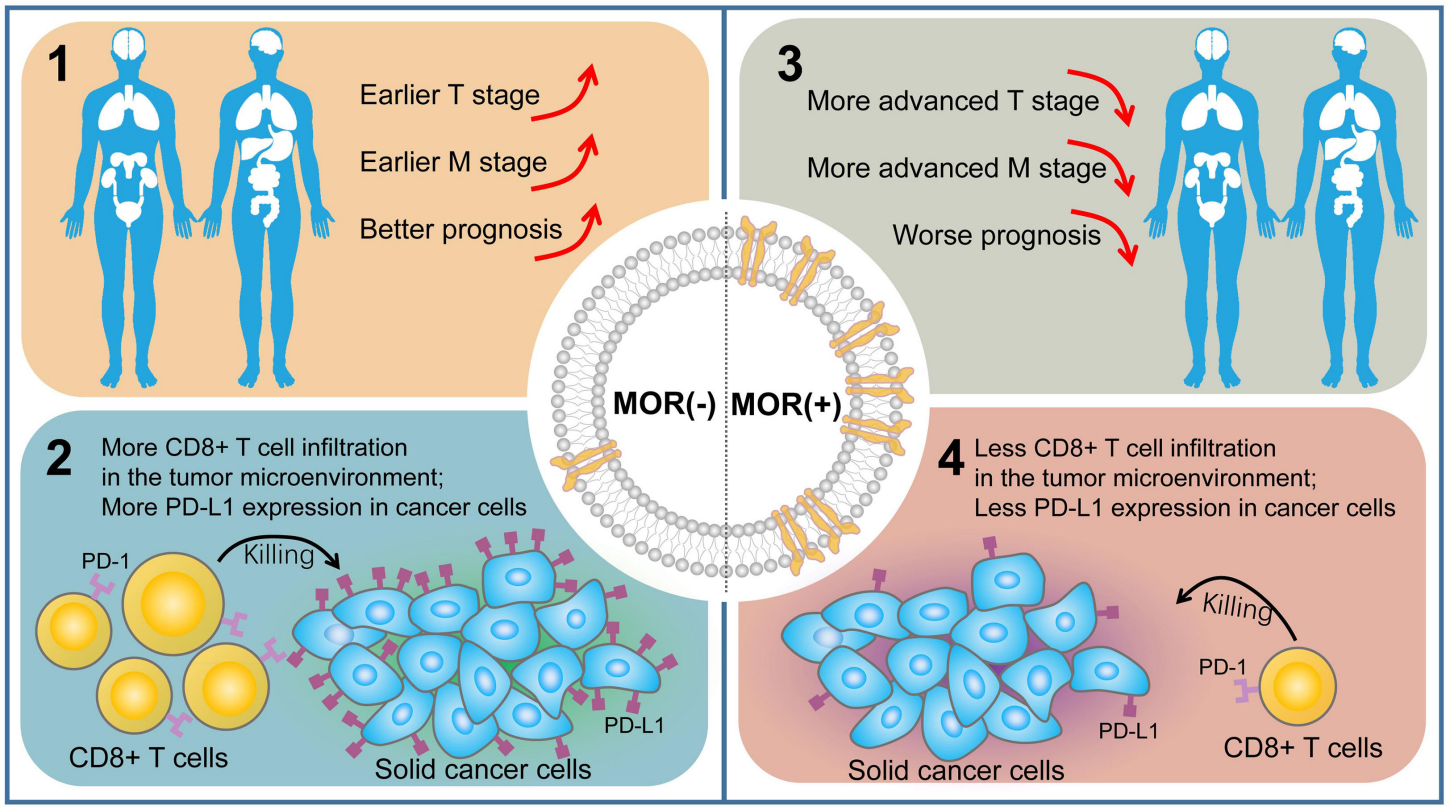
Summary of prognostic features and clinical implications of MOR.

To summarize, MOR mRNA overexpression in solid tumors was associated with advanced cancer and poor survival, which may be related to the tumor microenvironment (**Figure 4**). In the tumor microenvironment, increased MOR expression indicated decreased PD-L1 expression on cancer cells and decreased CD8+ T cell infiltrations, demonstrating poor response to PD-L1 therapy (**Figure 4**).

## 4 Discussion

In this study, we performed a pan-cancer analysis and found the prognostic features of *MOR* mRNA expression across 18 common solid cancer types. Firstly, we found that patients with positive *MOR* mRNA expression was associated with more advanced T and M stage. Secondly, *MOR* mRNA was identified as a prognostic biomarker in all cancer types. Positive *MOR* mRNA expression indicated worse prognosis for OS, PFS, DFS and DSS. Thirdly, patients with positive *MOR* mRNA expression tended to be classified as TMIT II, with low proportion of CD8+ CTLs and low PD-L1 expression, which implied poor response to PD-L1 therapies.

Opioids exert analgesic effects through acting on opioid receptors (mainly MOR) on neurons. Our study found that, out of a total of 7,274 patients, 1,112 were positive for *MOR* mRNA expression on the transcript level. When it comes to protein expression levels, a large number of laboratory studies have shown that cancer cells also expressed MOR, which were activated by opioids (18, 19). In line with our data, previous studies reported that MOR was upregulated in many cancers, and highly expressed MOR in cancers were correlated with cancer progression and recurrence (11, 20–22). A study of lung cancer also demonstrated the direct effect of opiates on cancer progression (23). The underlying molecular mechanisms remain elusive but it may be involved with the target receptors of opioids and downstream signaling pathways, induced by microRNAs’ modification (24). MOR overexpression promoted hepatocellular carcinoma cell proliferation and metastasis ability through EMT signaling pathway (11). MOR regulated self-renewal of hepatocellular carcinoma stem cells and acted as a potential therapeutic target *via* MOR-NFAT signaling pathway (19). Our study provides a potential explanation from the perspective of tumor immunology (25). Patients with refractory advanced cancer often receive anti-PD1/PDL1 therapy (26). However, not all solid tumors are sensitive to immunotherapy. The use of opioids is detrimental to survival outcomes for cancer patients receiving anti-PD-1/PD-L1 therapies (27). Two multicenter retrospective studies about solid cancers revealed that opioids used during immunotherapy were associated with a higher risk of early progression (28, 29), and shorter OS (30). Opioids may regulate immune cells in the tumor microenvironment, and then affect the tumor’s response to immunotherapy, including impairing T cell function and upregulating immunosuppressor cells (31), during which process MOR may serve as a potential target (29). For example, morphine-3-glucuronide upregulated PD-L1 expression through the PI3K signaling pathway, leading to the immune escape of non-small cell lung cancer cells (32). MOR may be used as a new biomarker for anti-PD1/PDL1 therapy sensitivity or not in patients with solid tumors (33).

Whether anesthesia influences cancer remains a key question in the field of anesthesia. Research on the relationship between opioid use and cancer outcomes is emerging (34, 35). MOR expression is associated with opioid use. Intraoperative opioid use increases MOR expression in cancer tissues (36, 37), and also exacerbates shorter survival (38). Contrarily, sufentanil consumption was higher in the MOR high expression group (21, 39). Generally, opioid administration may promote cancer progression, recurrence and reduce survival. For patients with non-small-cell lung carcinoma, increased doses of opioids during the postoperative period were associated with a higher 5-year recurrence rate (8). The consumption of opioids is increasing to manage chronic cancer pain in Western countries (40). While greater opioid requirement for advanced cancer pain was independently associated with reduced survival in advanced non-small cell lung cancer (41). However, a Danish population-based study found no association between opioid prescriptions and recurrence in breast cancer (42). There was also no association between postoperative opioid consumption and cancer progression or all-cause mortality in surgical patients with colorectal cancer according to another retrospective cohort study (43).

The impacts of opioids on cancer outcome vary from cancers to cancers. Certain types of cancer are sensitive to opioids, in which opioid consumption may lead to worse survival of patients. Our study may explain such differences. Our data showed that the correlation between *MOR* mRNA expression and poor prognosis is strong in BRCA, LIHC, lung adenocarcinoma (LUAD) and LUSC, which was consistent with previous studies (10, 11, 42). In contrast, the predictive outcome of *MOR* also exists in other cancer types although relatively weak (44). Interestingly, prostate adenocarcinoma (PRAD) patients with *MOR* mRNA expression have a better prognosis, which is rarely reported yet (45) but warrants further study.

Among patients of solid cancers, increased *MOR* mRNA expression was associated with reduced survival, also accompanied by advanced-stage cancer. In the tumor microenvironment, increased *MOR* expression indicated less PD-L1 expression in cancer cells, as well as less CD8+ T cell infiltrations, which responded poorly to immunotherapy. Our research is of practical significance. Although opioid-based analgesia is necessary for controlling cancer pain, the long-term opioid use may activate oncogenic pathways and lead to a worse prognosis (46). Therefore, use alternative analgesics including local and regional block for cancer relief is urgently needed to avoid its side effects including addiction (47); use intrathecal opioid pump or multimodal analgesia to reduce the use of opioids are strongly recommended (48, 49).

There were still some limitations in our study. Firstly, some patients’ clinicopathological data is missing in the TCGA database and therefore, our study may not reflect patient population. Secondly, it is a retrospective study and causal relationship of *MOR and* cancer outcomes is still unknown. Thirdly, the data were transcriptome in our study, further transcriptome, proteome or even epigenetic data analysis are also needed in future studies.

In conclusion, this is the first pan-cancer study revealing the prognostic role of *MOR* mRNA expression across 18 cancers. Our data showed that most tumors commonly express *MOR* mRNA. *MOR* mRNA was a prognostic biomarker cross all cancer types studied. Our work may indicate *MOR* mRNA overexpression in solid cancer represented poor prognosis and responded poorly to PD-L1 therapy although warrants future study.

## Data availability statement

Publicly available datasets were analyzed in this study. This data can be found here: TCGA database.

## Author contributions

WS, SZ, MC and ZQ were responsible for the conception and design of the study, drafting and writing of the article, acquisition and analysis of data. WS and ZQ were responsible for the interpretation of the data and drawing the figures. All authors contributed to the article and approved the submitted version.
