# Supplementary material for: Mu opioid receptor mRNA overexpression predicts poor prognosis among 18 common solid cancers: A pan-cancer analysis
Source: Front Oncol. 2023 Mar 30;13:1134744. doi: 10.3389/fonc.2023.1134744 (PMC10098160; doi:10.3389/fonc.2023.1134744)
Supplement: Supplementary file 1 [file DataSheet_1.pdf]

Supplementary Material

1.1 Supplementary Figures

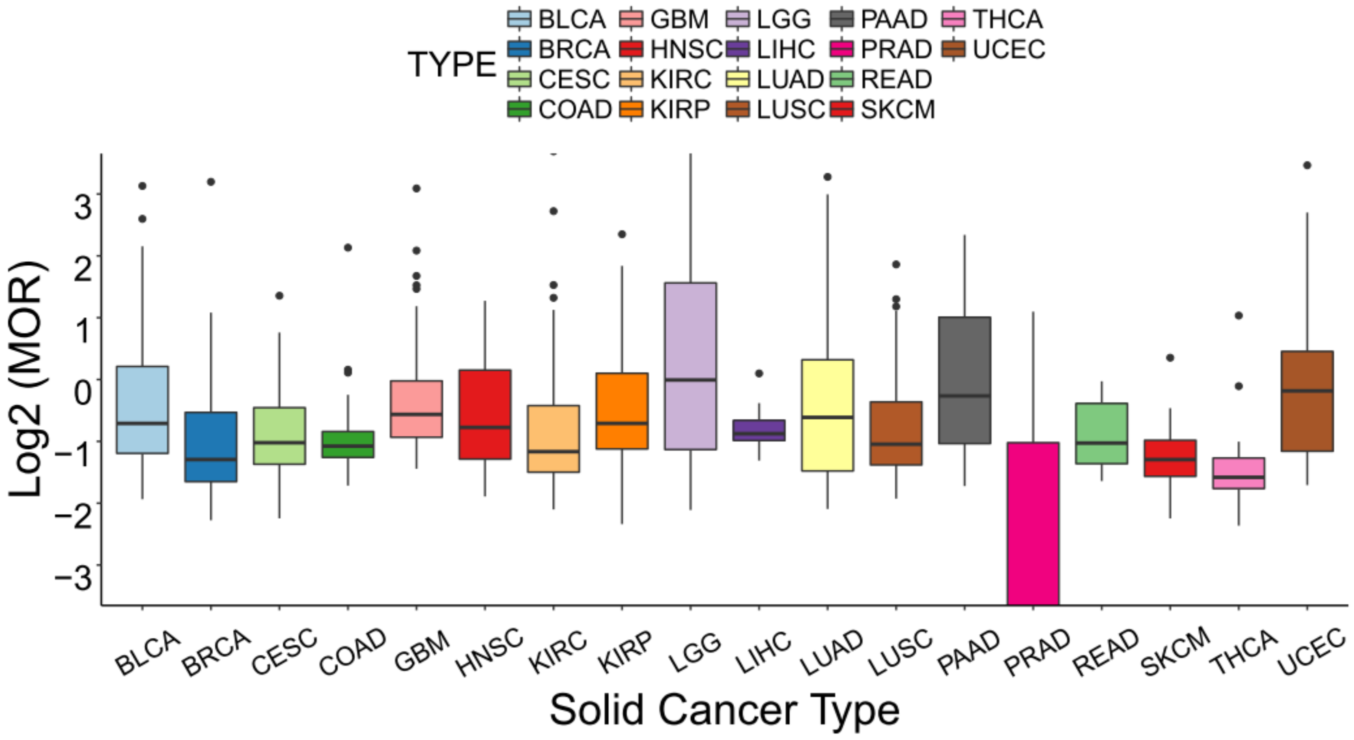

Supplementary Figure 1. Distribution of *MOR* mRNA expression across 18 cancer types.

Footnotes: The boxplot displays log 2 conversion values of *MOR* mRNA expression.

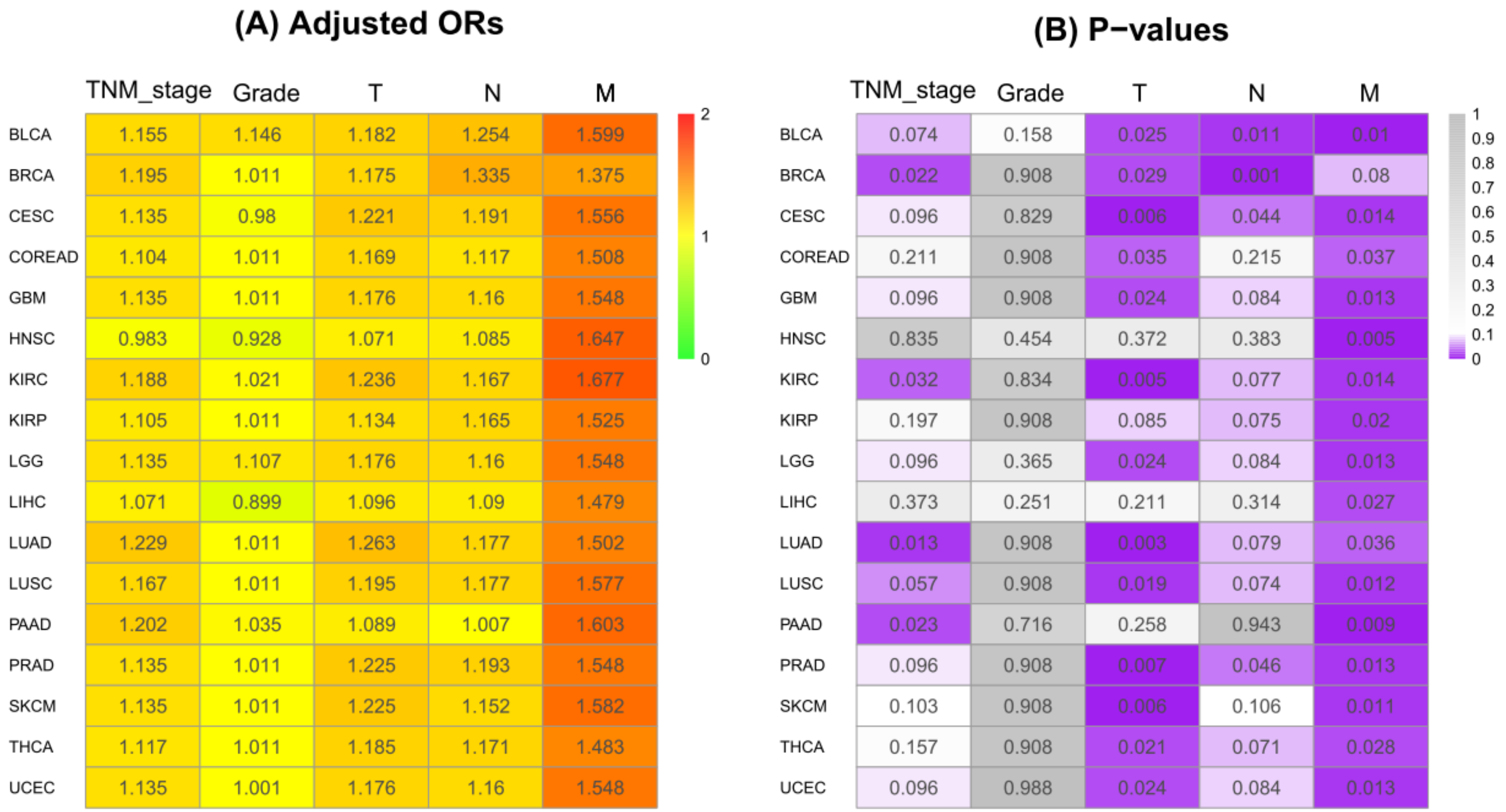

Supplementary Figure 2. Association between clinicopathological features and *MOR* mRNA expression for sensitivity analysis.

Footnotes: The heatmaps show (A) adjusted ORs and (B) *P*-values between clinicopathological features (AJCC stage, grade, T stage, N stage and M stage) and *MOR* mRNA expression for sensitivity analysis. Different colours correspond to different values from small to large.

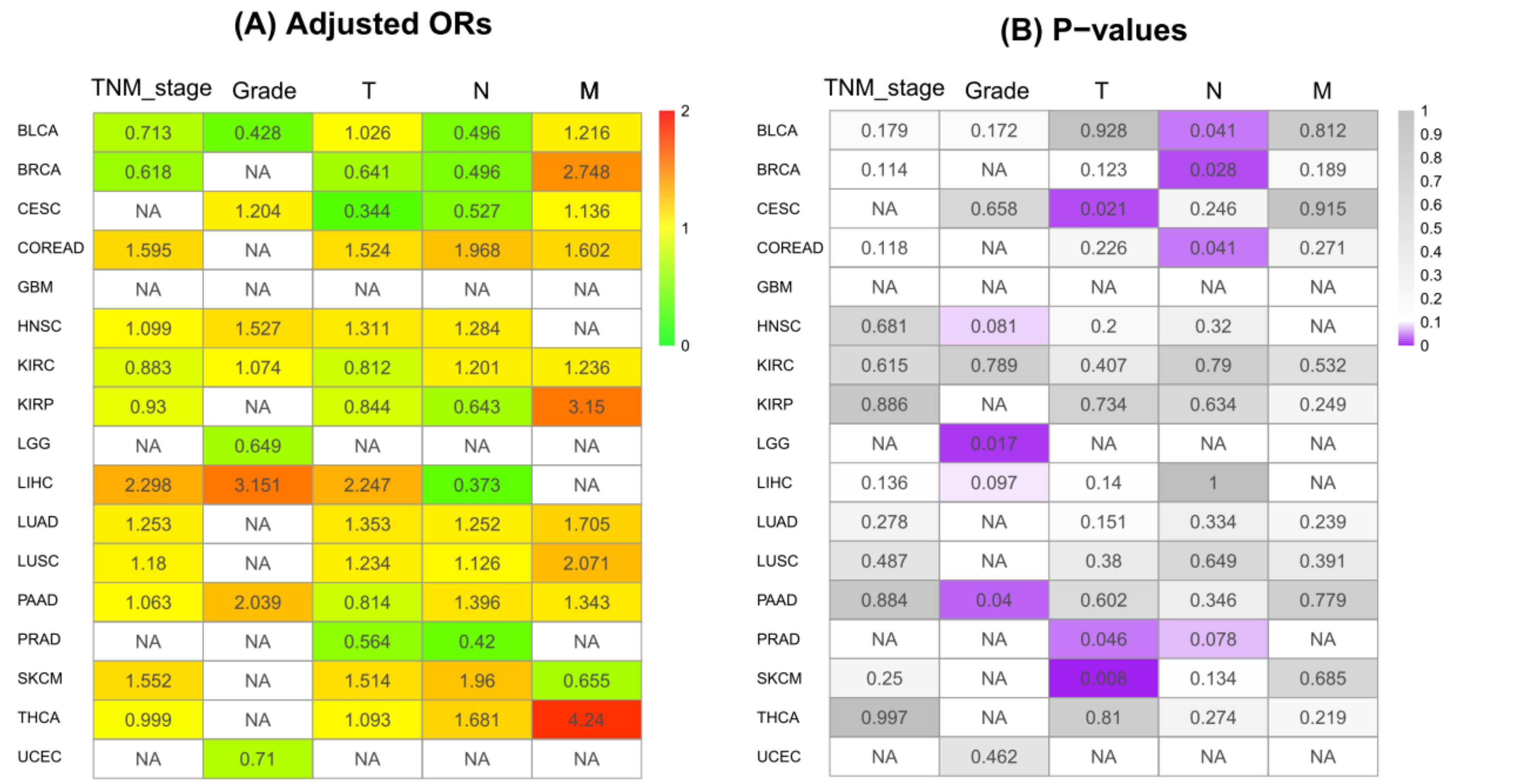

Supplementary Figure 3. Association between clinicopathological features and *MOR* mRNA expression across different cancer types for subgroup analysis.

Footnotes: The heatmaps show (A) adjusted ORs and (B) *P*-values between clinicopathological features (AJCC stage, grade, T stage, N stage and M stage) and *MOR* mRNA expression in each cancer type. Different colors correspond to different values from small to large.

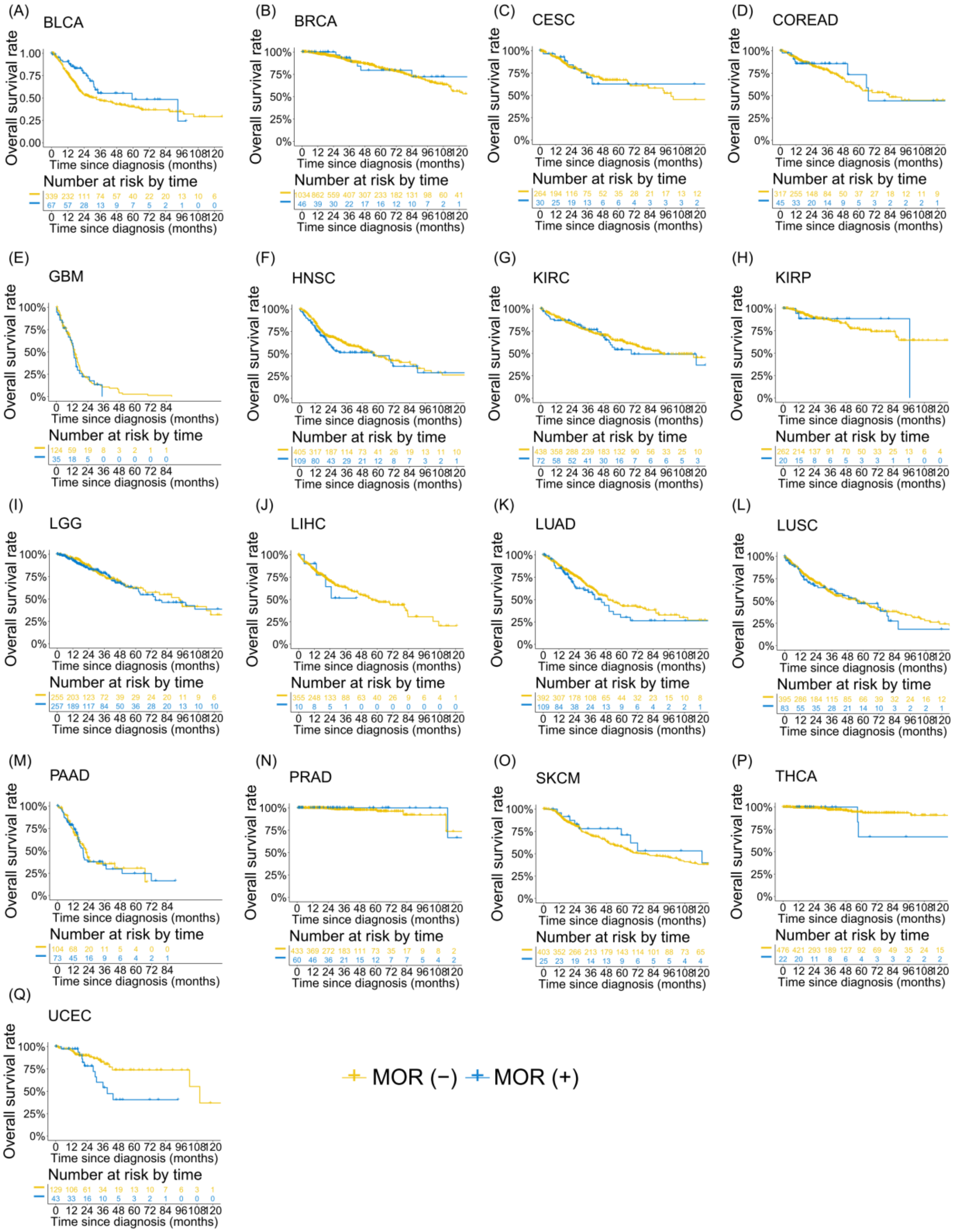

Supplementary Figure 4. Kaplan-Meier curves for OS according to *MOR* mRNA expression across different cancer types.

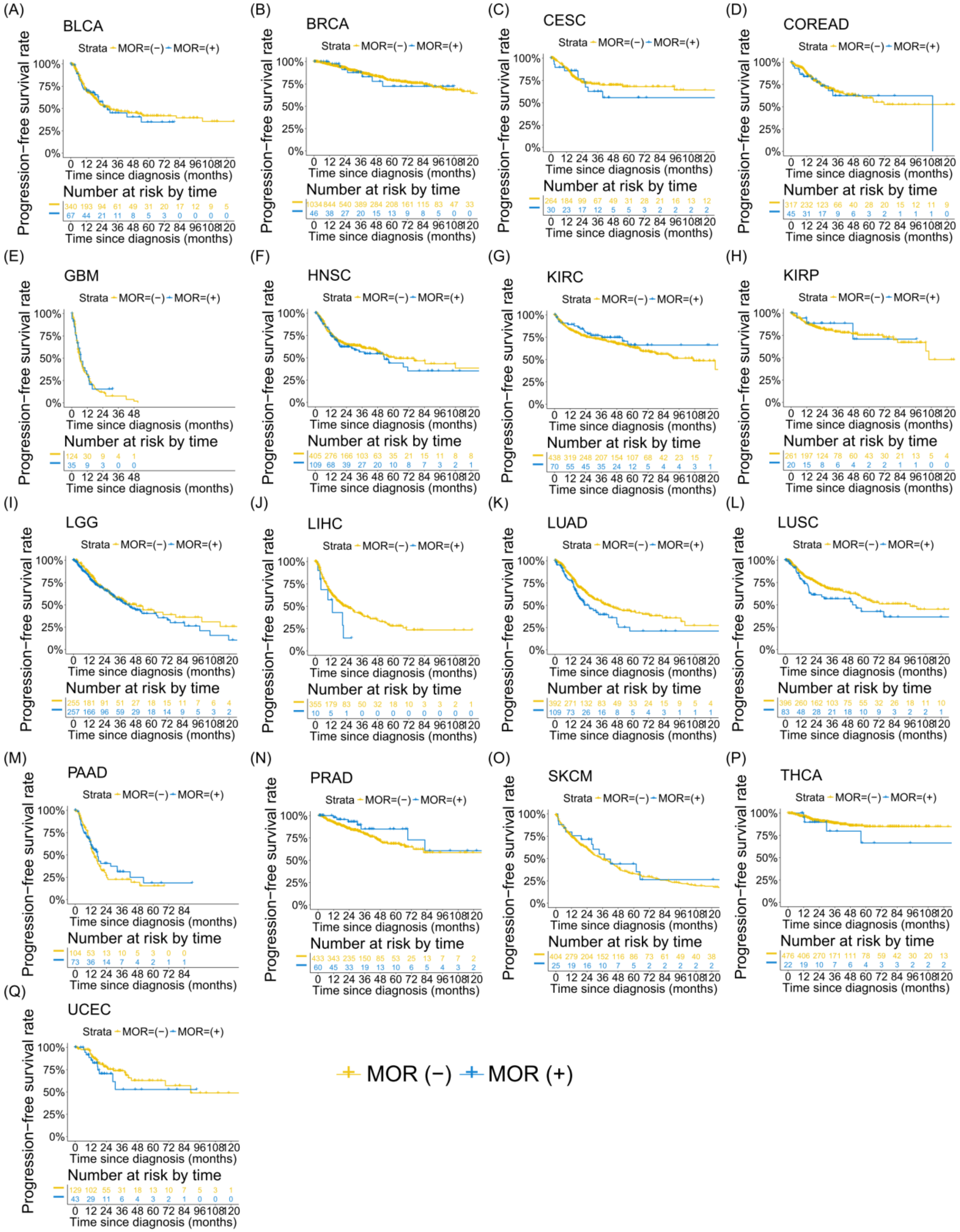

Supplementary Figure 5. Kaplan-Meier curves for PFS according to *MOR* mRNA expression across different cancer types.

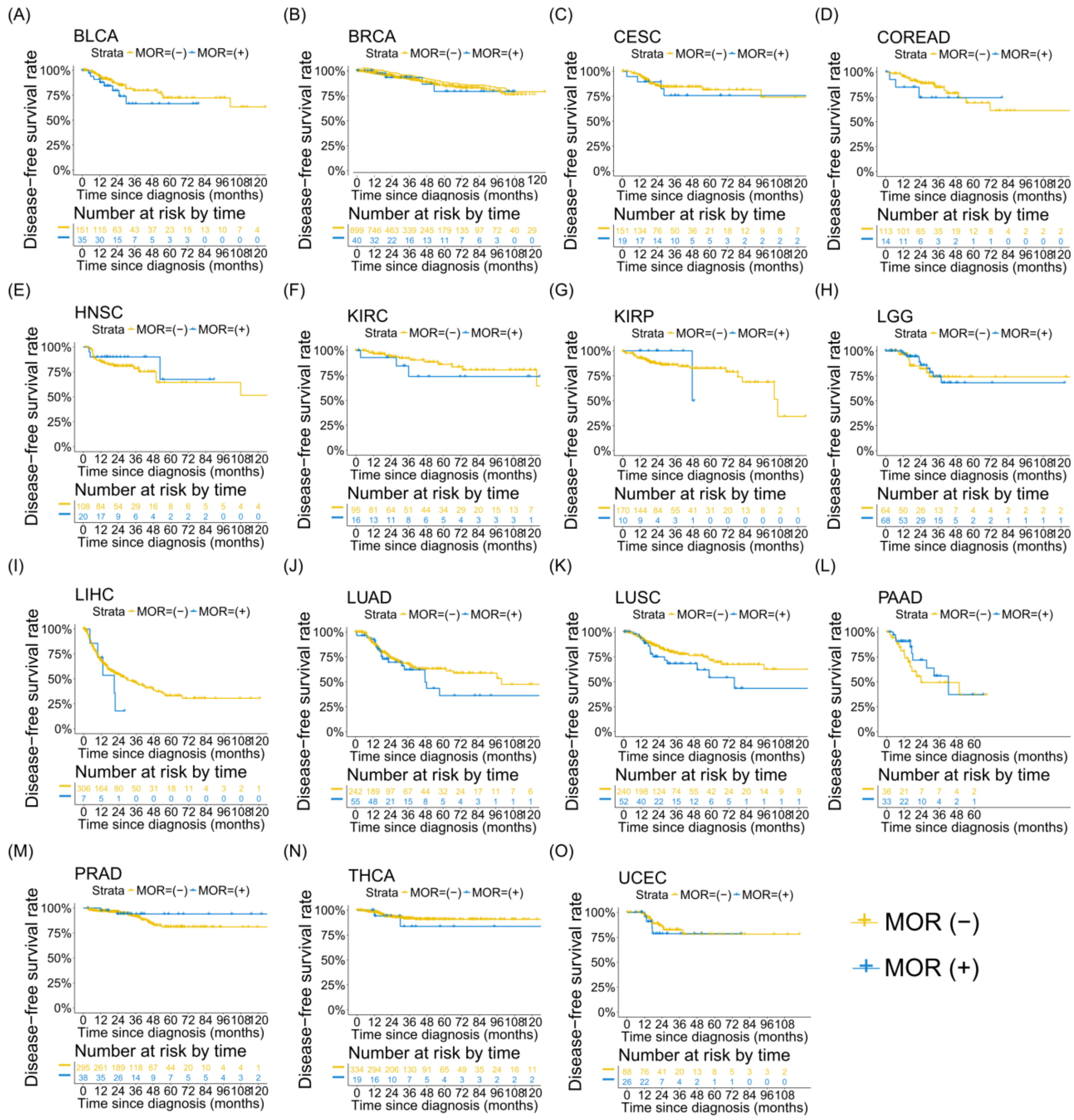

Supplementary Figure 6. Kaplan-Meier curves for DFS according to *MOR* mRNA expression across different cancer types.

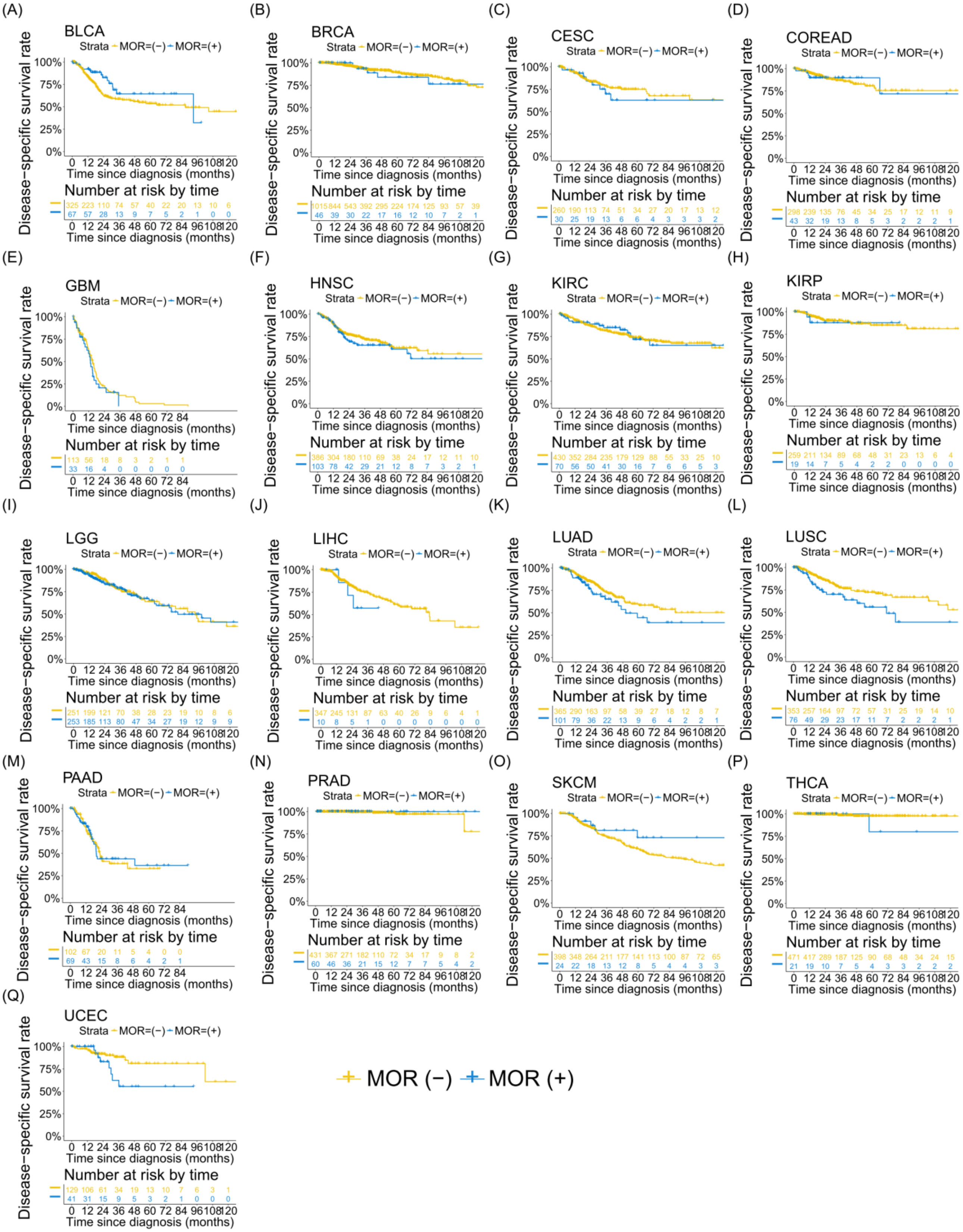

Supplementary Figure 7. Kaplan-Meier curves for DSS according to *MOR* mRNA expression across different cancer types.

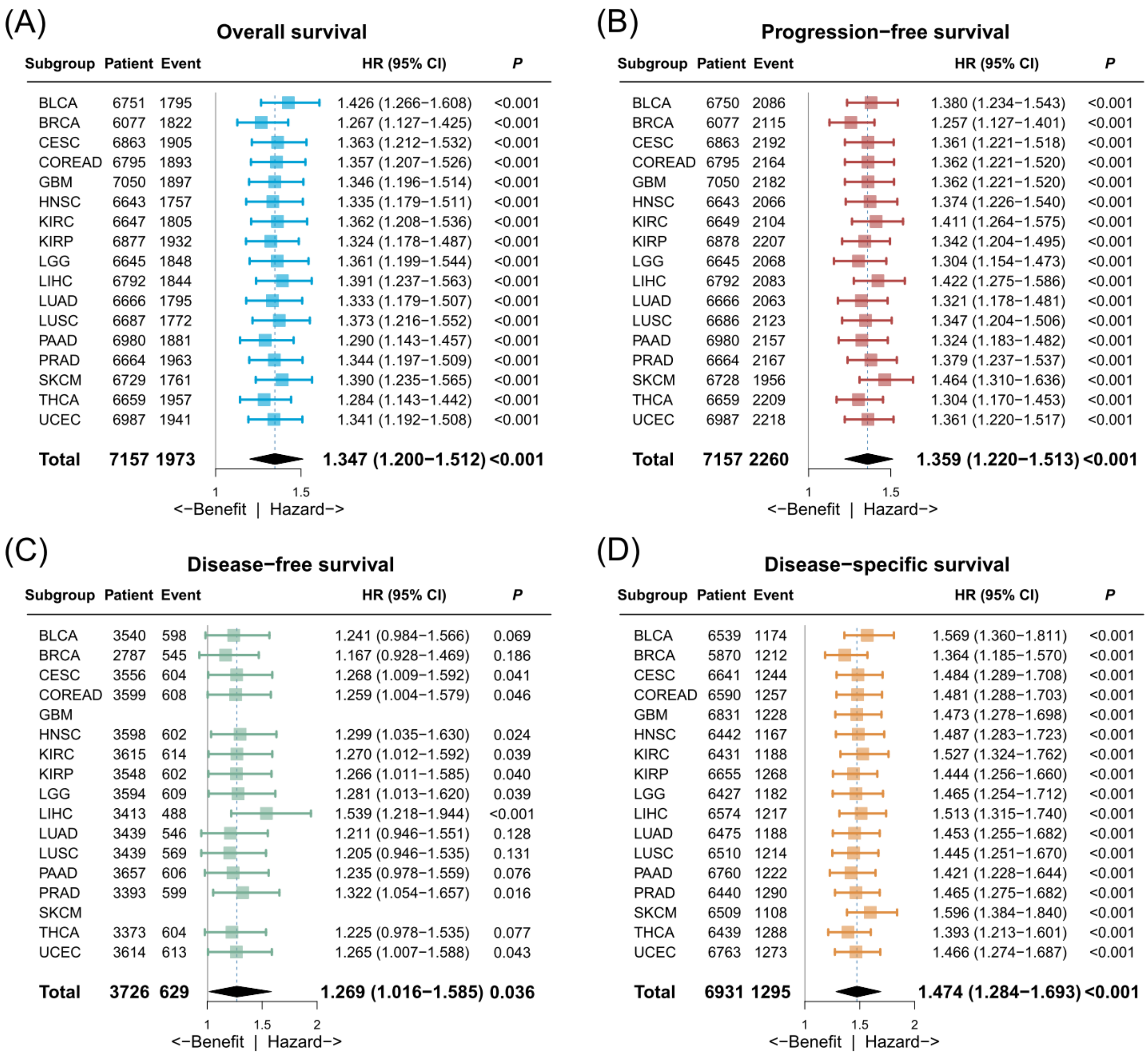

Supplementary Figure 8. The impact of *MOR* mRNA expression on prognosis for sensitivity analysis.

Footnotes: Forest map for (A) OS, (B) PFS, (C) DFS and (D) DSS.

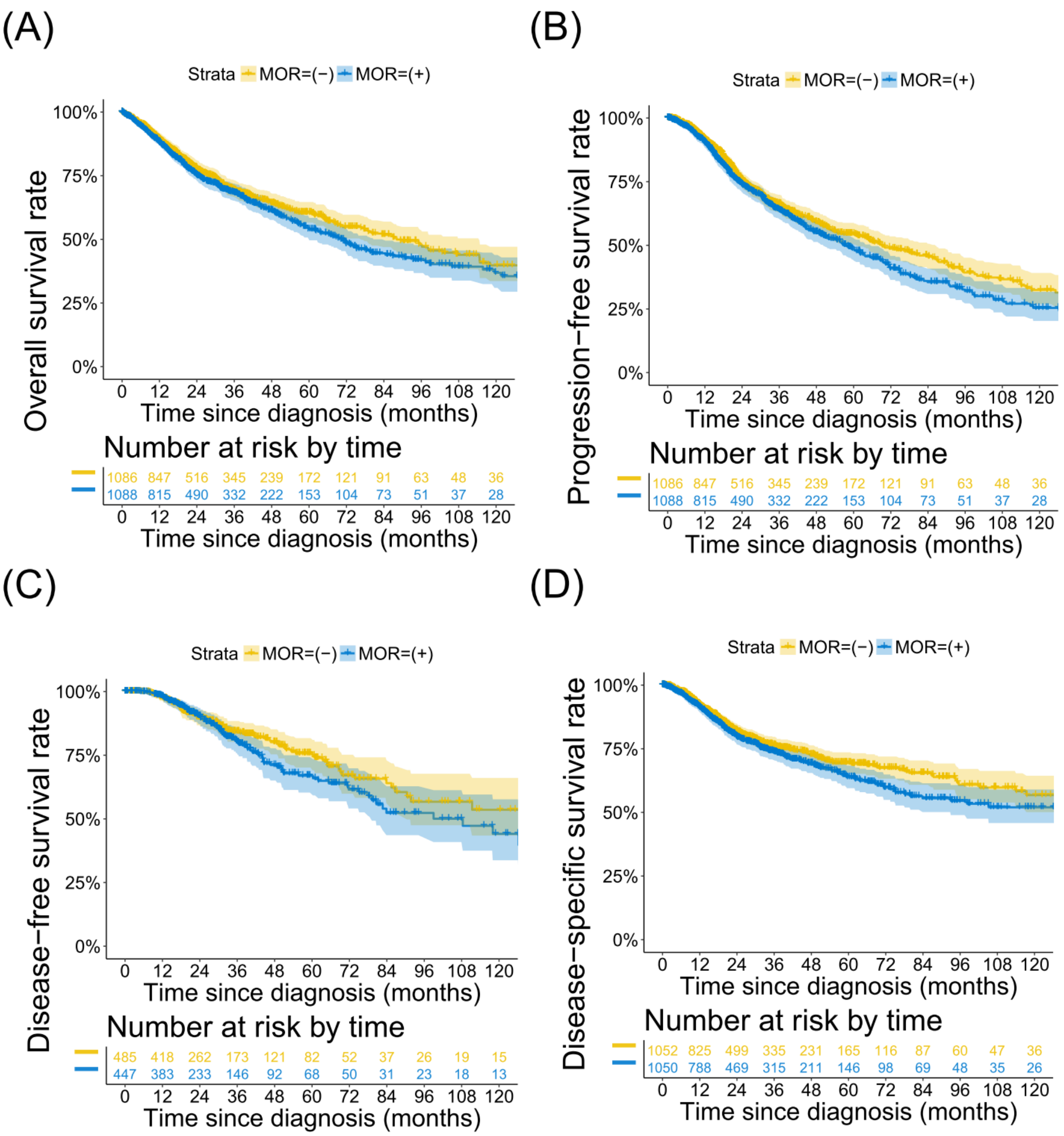

Supplementary Figure 9. Kaplan-Meier survival curves according to *MOR* mRNA expression with the balanced data after PSM.

Footnotes: Kaplan-Meier curves for (A) OS, (B) PFS, (C) DFS and (D) DSS.

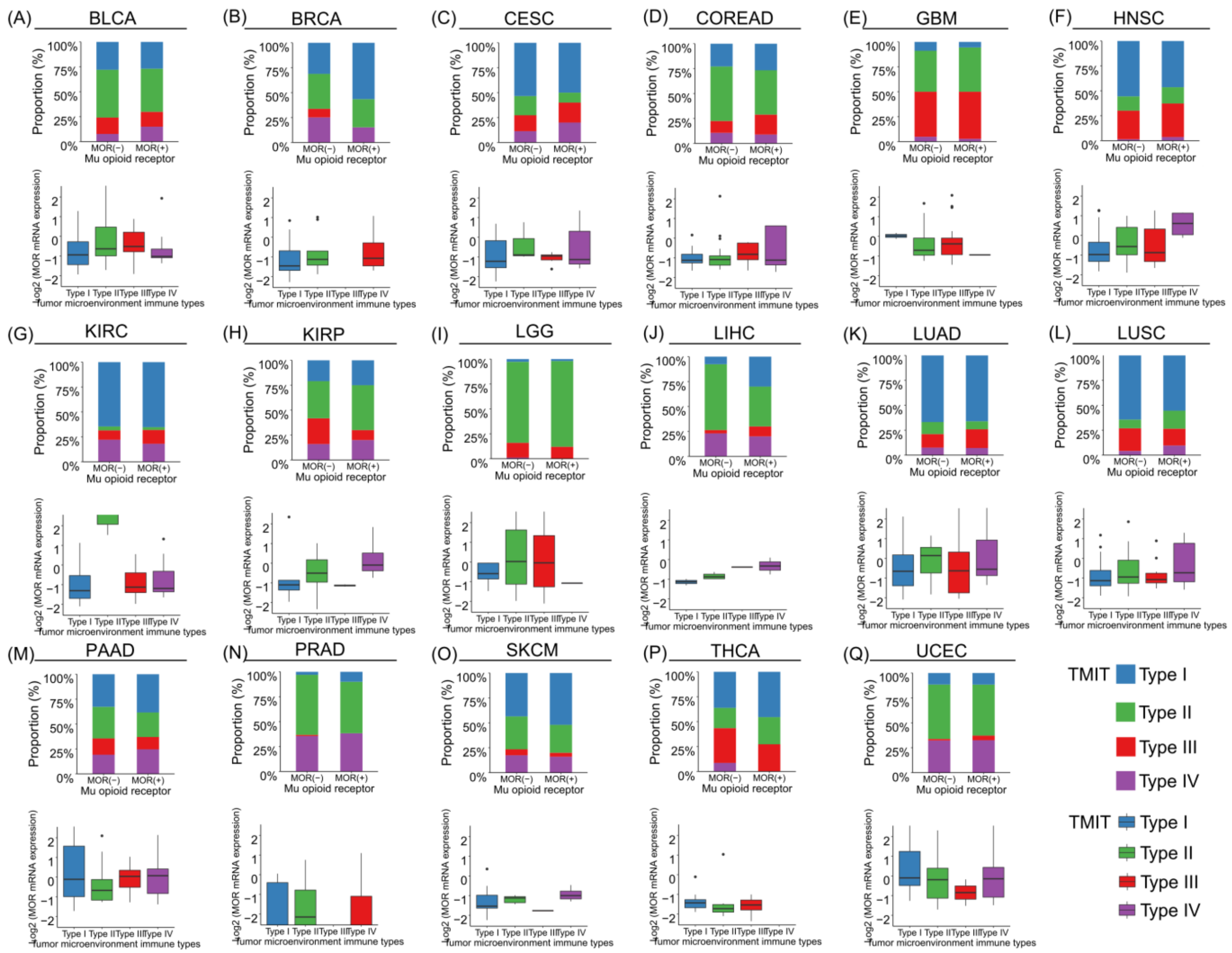

Supplementary Figure 10. Distribution of TMITs according to *MOR* mRNA expression, and *MOR* mRNA expression according to TMIT across different cancer types.

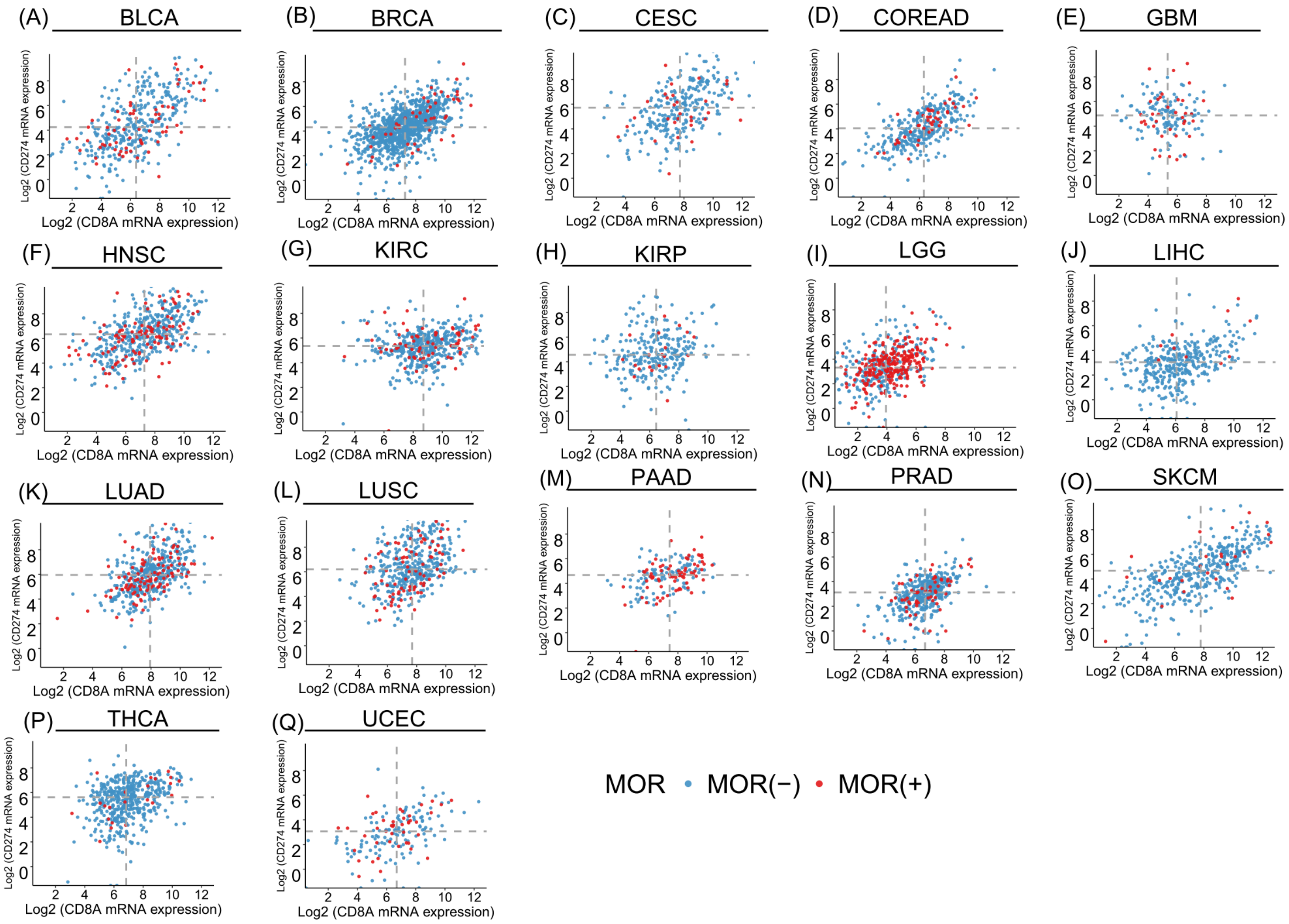

Supplementary Figure 11. Correlation between mRNA expression level of PD-L1 and CD8A across different cancer types.

Footnotes: Blue dots indicate patients with negative *MOR* mRNA expression; Red dots indicate patients with positive *MOR* mRNA expression; Grey dotted lines indicate the mean expression.

1.2 Supplementary Tables

Supplementary Table 1. Baseline characteristics of included patients by cancer type.

Characteristic		BLCA (407)	BRCA(1082)	CESC(294)	COAD(276)	GBM(160)	HNSC(515)	KIRC(510)	KIRP(283)	LGG(514)
MOR	Positive	67 (16.5)	46 (4.3)	30 (10.2)	28 (10.1)	36 (22.5)	110 (21.4)	72 (14.1)	20 (7.1)	258 (50.2)
	Negative	340 (83.5)	1036 (95.7)	264 (89.8)	248 (89.9)	124 (77.5)	405 (78.6)	438 (85.9)	263 (92.9)	256 (49.8)
Age		68.1±10.6	58.4±13.2	48.2±13.9	65.0±13.3	60.2±14.0	60.8±11.8	60.5±12.1	61.4±12.1	42.9±13.4
Age group	Young	106 (26.0)	598 (55.3)	237 (80.6)	99 (35.9)	55 (34.4)	255 (49.5)	256 (50.2)	131 (46.3)	452 (87.9)
	Old	301 (74.0)	484 (44.7)	57 (19.4)	175 (63.4)	52 (32.5)	259 (50.3)	254 (49.8)	149 (52.7)	61 (11.9)
	Unknown	0 (0.0)	0 (0.0)	0 (0.0)	2 (0.7)	53 (33.1)	1 (0.2)	0 (0.0)	3 (1.1)	1 (0.2)
Gender	Female	107 (26.3)	1070 (98.9)	294 (100.0)	122 (44.2)	44 (27.5)	135 (26.2)	185 (36.3)	72 (25.4)	228 (44.4)
	Male	300 (73.7)	12 (1.1)	0 (0.0)	152 (55.1)	63 (39.4)	380 (73.8)	325 (63.7)	211 (74.6)	285 (55.4)
	Unknown	0 (0.0)	0 (0.0)	0 (0.0)	2 (0.7)	53 (33.1)	0 (0.0)	0 (0.0)	0 (0.0)	1 (0.2)
Race	Caucasian	324 (79.6)	749 (69.2)	201 (68.4)	188 (68.1)	96 (60.0)	440 (85.4)	440 (86.3)	199 (70.3)	474 (92.2)
	African	23 (5.7)	182 (16.8)	28 (9.5)	55 (19.9)	6 (3.8)	47 (9.1)	55 (10.8)	61 (21.6)	21 (4.1)
	Others	60 (14.7)	151 (14.0)	65 (22.1)	33 (12.0)	58 (36.2)	28 (5.4)	15 (2.9)	23 (8.1)	19 (3.7)
AJCC stage	Stage I	2 (0.5)	180 (16.6)	0 (0.0)	45 (16.3)	0 (0.0)	27 (5.2)	249 (48.8)	169 (59.7)	0 (0.0)
	Stage II	129 (31.7)	615 (56.8)	0 (0.0)	107 (38.8)	0 (0.0)	74 (14.4)	54 (10.6)	22 (7.8)	0 (0.0)
	Stage III	140 (34.4)	249 (23.0)	0 (0.0)	79 (28.6)	0 (0.0)	79 (15.3)	124 (24.3)	50 (17.7)	0 (0.0)
	Stage IV	134 (32.9)	19 (1.8)	0 (0.0)	36 (13.0)	0 (0.0)	265 (51.5)	83 (16.3)	15 (5.3)	0 (0.0)
	Others	2 (0.5)	19 (1.8)	294 (100.0)	9 (3.3)	160 (100.0)	70 (13.6)	0 (0.0)	27 (9.5)	514 (100.0)
Grade	Low Grade	21 (5.2)	0 (0.0)	147 (50.0)	0 (0.0)	0 (0.0)	363 (70.5)	227 (44.5)	0 (0.0)	248 (48.2)

	High Grade	383 (94.1)	0 (0.0)	115 (39.1)	0 (0.0)	0 (0.0)	130 (25.2)	275 (53.9)	0 (0.0)	264 (51.4)
	Others	3 (0.7)	1082 (100.0)	32 (10.9)	276 (100.0)	160 (100.0)	22 (4.3)	8 (1.6)	283 (100.0)	2 (0.4)
AJCC-T	T1	3 (0.7)	276 (25.5)	134 (45.6)	6 (2.2)	0 (0.0)	48 (9.3)	254 (49.8)	189 (66.8)	0 (0.0)
	T2	118 (29.0)	627 (57.9)	71 (24.1)	43 (15.6)	0 (0.0)	134 (26.0)	66 (12.9)	32 (11.3)	0 (0.0)
	T3	194 (47.7)	137 (12.7)	20 (6.8)	187 (67.8)	0 (0.0)	97 (18.8)	179 (35.1)	58 (20.5)	0 (0.0)
	T4	58 (14.3)	36 (3.3)	8 (2.7)	37 (13.4)	0 (0.0)	173 (33.6)	11 (2.2)	2 (0.7)	0 (0.0)
	Others	34 (8.4)	6 (0.6)	61 (20.7)	3 (1.1)	160 (100.0)	63 (12.2)	0 (0.0)	2 (0.7)	514 (100.0)
AJCC-N	N0	236 (58.0)	512 (47.3)	129 (43.9)	162 (58.7)	0 (0.0)	174 (33.8)	228 (44.7)	47 (16.6)	0 (0.0)
	N1	46 (11.3)	355 (32.8)	57 (19.4)	68 (24.6)	0 (0.0)	66 (12.8)	16 (3.1)	24 (8.5)	0 (0.0)
	N2	75 (18.4)	119 (11.0)	0 (0.0)	44 (15.9)	0 (0.0)	168 (32.6)	0 (0.0)	4 (1.4)	0 (0.0)
	N3	8 (2.0)	76 (7.0)	0 (0.0)	0 (0.0)	0 (0.0)	8 (1.6)	0 (0.0)	0 (0.0)	0 (0.0)
	Others	42 (10.3)	20 (1.8)	108 (36.7)	2 (0.7)	160 (100.0)	99 (19.2)	266 (52.2)	208 (73.5)	514 (100.0)
AJCC-M	M0	195 (47.9)	900 (83.2)	111 (37.8)	184 (66.7)	0 (0.0)	183 (35.5)	401 (78.6)	91 (32.2)	0 (0.0)
	M1	11 (2.7)	21 (1.9)	9 (3.1)	36 (13.0)	0 (0.0)	1 (0.2)	78 (15.3)	9 (3.2)	0 (0.0)
	Others	201 (49.4)	161 (14.9)	174 (59.2)	56 (20.3)	160 (100.0)	331 (64.3)	31 (6.1)	183 (64.7)	514 (100.0)

Characteristic		LIHC(366)	LUAD(510)	LUSC(484)	PAAD(177)	PRAD(493)	READ(89)	SKCM(443)	THCA(498)	UCEC(173)
MOR	Positive	10 (2.7)	112 (22.0)	83 (17.1)	73 (41.2)	60 (12.2)	17 (19.1)	25 (5.6)	22 (4.4)	43 (24.9)
	Negative	356 (97.3)	398 (78.0)	401 (82.9)	104 (58.8)	433 (87.8)	72 (80.9)	418 (94.4)	476 (95.6)	130 (75.1)
Age		59.5±13.4	65.3±10.0	67.2±8.5	64.5±10.9	61.0±6.9	62.7±12.6	57.5±15.7	47.3±15.8	65.7±11.4
Age group	Young	175 (47.8)	157 (30.8)	103 (21.3)	59 (33.3)	221 (44.8)	40 (44.9)	245 (55.3)	387 (77.7)	54 (31.2)
	Old	190 (51.9)	334 (65.5)	370 (76.4)	118 (66.7)	272 (55.2)	49 (55.1)	190 (42.9)	111 (22.3)	116 (67.1)
	Unknown	1 (0.3)	19 (3.7)	11 (2.3)	0 (0.0)	0 (0.0)	0 (0.0)	8 (1.8)	0 (0.0)	3 (1.7)

Gender	Female	120 (32.8)	274 (53.7)	126 (26.0)	80 (45.2)	0 (0.0)	42 (47.2)	169 (38.1)	363 (72.9)	173 (100.0)
	Male	246 (67.2)	236 (46.3)	356 (73.6)	97 (54.8)	493 (100.0)	47 (52.8)	274 (61.9)	135 (27.1)	0 (0.0)
	Unknown	0 (0.0)	0 (0.0)	2 (0.4)	0 (0.0)	0 (0.0)	0 (0.0)	0 (0.0)	0 (0.0)	0 (0.0)
Race	Caucasian	180 (49.2)	384 (75.3)	335 (69.2)	156 (88.1)	147 (29.8)	73 (82.0)	424 (95.7)	329 (66.1)	83 (48.0)
	African	17 (4.6)	52 (10.2)	28 (5.8)	6 (3.4)	7 (1.4)	5 (5.6)	1 (0.2)	27 (5.4)	65 (37.6)
	Others	169 (46.2)	74 (14.5)	121 (25.0)	15 (8.5)	339 (68.8)	11 (12.4)	18 (4.1)	142 (28.5)	25 (14.5)
AJCC stage	Stage I	169 (46.2)	275 (53.9)	235 (48.6)	21 (11.9)	0 (0.0)	12 (13.5)	89 (20.1)	282 (56.6)	0 (0.0)
	Stage II	86 (23.5)	123 (24.1)	157 (32.4)	146 (82.5)	0 (0.0)	27 (30.3)	119 (26.9)	51 (10.2)	0 (0.0)
	Stage III	84 (23.0)	83 (16.3)	82 (16.9)	4 (2.3)	0 (0.0)	31 (34.8)	167 (37.7)	109 (21.9)	0 (0.0)
	Stage IV	6 (1.6)	27 (5.3)	7 (1.4)	4 (2.3)	0 (0.0)	15 (16.9)	24 (5.4)	54 (10.8)	0 (0.0)
	Others	21 (5.7)	2 (0.4)	3 (0.6)	2 (1.1)	493 (100.0)	4 (4.5)	44 (9.9)	2 (0.4)	173 (100.0)
Grade	Low Grade	227 (62.0)	0 (0.0)	0 (0.0)	125 (70.6)	0 (0.0)	0 (0.0)	0 (0.0)	0 (0.0)	34 (19.7)
	High Grade	134 (36.6)	0 (0.0)	0 (0.0)	50 (28.2)	0 (0.0)	0 (0.0)	0 (0.0)	0 (0.0)	139 (80.3)
	Others	5 (1.4)	510 (100.0)	484 (100.0)	2 (1.1)	493 (100.0)	89 (100.0)	443 (100.0)	498 (100.0)	0 (0.0)
AJCC-T	T1	178 (48.6)	167 (32.7)	107 (22.1)	7 (4.0)	0 (0.0)	4 (4.5)	41 (9.3)	142 (28.5)	0 (0.0)
	T2	93 (25.4)	275 (53.9)	282 (58.3)	23 (13.0)	186 (37.7)	13 (14.6)	77 (17.4)	165 (33.1)	0 (0.0)
	T3	79 (21.6)	46 (9.0)	70 (14.5)	142 (80.2)	290 (58.8)	62 (69.7)	89 (20.1)	167 (33.5)	0 (0.0)
	T4	13 (3.6)	19 (3.7)	23 (4.8)	3 (1.7)	10 (2.0)	10 (11.2)	129 (29.1)	22 (4.4)	0 (0.0)
	Others	3 (0.8)	3 (0.6)	2 (0.4)	2 (1.1)	7 (1.4)	0 (0.0)	107 (24.2)	2 (0.4)	173 (100.0)
AJCC-N	N0	251 (68.6)	328 (64.3)	306 (63.2)	49 (27.7)	342 (69.4)	41 (46.1)	216 (48.8)	226 (45.4)	0 (0.0)
	N1	3 (0.8)	96 (18.8)	126 (26.0)	123 (69.5)	78 (15.8)	27 (30.3)	73 (16.5)	222 (44.6)	0 (0.0)
	N2	0 (0.0)	73 (14.3)	39 (8.1)	0 (0.0)	0 (0.0)	20 (22.5)	47 (10.6)	0 (0.0)	0 (0.0)

	N3	0 (0.0)	2 (0.4)	5 (1.0)	0 (0.0)	0 (0.0)	0 (0.0)	55 (12.4)	0 (0.0)	0 (0.0)
	Others	112 (30.6)	11 (2.2)	8 (1.7)	5 (2.8)	73 (14.8)	1 (1.1)	52 (11.7)	50 (10.0)	173 (100.0)
AJCC-M	M0	263 (71.9)	341 (66.9)	392 (81.0)	79 (44.6)	0 (0.0)	65 (73.0)	390 (88.0)	277 (55.6)	0 (0.0)
	M1	4 (1.1)	25 (4.9)	7 (1.4)	4 (2.3)	0 (0.0)	12 (13.5)	25 (5.6)	9 (1.8)	0 (0.0)
	Others	99 (27.0)	144 (28.2)	85 (17.6)	94 (53.1)	493 (100.0)	12 (13.5)	28 (6.3)	212 (42.6)	173 (100.0)

Footnotes: Young, under 60 years old; Old, over 60 years old; All the variables were described as frequencies and percentages, except for age described as mean and standard deviation.

Abbreviations: BLCA, bladder urothelial carcinoma; BRCA, breast invasive carcinoma; CESC, cervical squamous cell carcinoma; COAD, colon adenocarcinoma; GBM, glioblastoma multiforme; HNSC, head and neck squamous cell carcinoma; KIRC, kidney renal clear cell carcinoma; KIRP, kidney renal papillary cell carcinoma; LGG, brain lower grade Glioma; LIHC, liver hepatocellular carcinoma; LUAD, lung adenocarcinoma; LUSC, lung squamous cell carcinoma; PAAD, pancreatic adenocarcinoma; PRAD, prostate adenocarcinoma; READ, rectal adenocarcinoma; SKCM, skin cutaneous melanoma; THCA, thyroid carcinoma; UCEC, uterine corpus endometrial carcinoma; MOR, mu opioid receptor; TCGA, the Cancer Genome Atlas; AJCC, the American Joint Committee on Cancer; TNM, Tumor node metastasis.

Supplementary Table 2. Baseline characteristics of the included patients in the TCGA database.

Characteristic	Total<br>N = 2178	*MOR* (-)<br>N = 1089	*MOR* (+)<br>N = 1089	*P*-value
Age				0.687
	57.6±15.1	57.7±15.0	57.4±15.2	
Age group				0.999
Young	1143	572 (52.5)	571 (52.4)	
Old	1035	517 (47.5)	518 (47.6)	
Gender				0.547
Female	987	501 (46.0)	486 (44.6)	
Male	1191	588 (54.0)	603 (55.4)	
Race				0.792
Caucasian	1702	856 (78.6)	846 (77.7)	
African	201	96 (8.8)	105 (9.6)	
Others	275	137 (12.6)	138 (12.7)	
AJCC stage				0.972
Stage I	391	197 (18.1)	194 (17.8)	
Stage II	417	204 (18.7)	213 (19.6)	
Stage III	264	129 (11.8)	135 (12.4)	
Stage IV	236	118 (10.8)	118 (10.8)	
Others	870	441 (40.5)	429 (39.4)	
Grade				0.904
Low Grade	633	315 (28.9)	318 (29.2)	
High Grade	657	325 (29.8)	332 (30.5)	
Others	888	449 (41.2)	439 (40.3)	
AJCC-T				0.995
T1	301	150 (13.8)	151 (13.9)	
T2	547	272 (25.0)	275 (25.3)	
T3	430	212 (19.5)	218 (20.0)	
T4	184	93 (8.5)	91 (8.4)	
Others	716	362 (33.2)	354 (32.5)	
AJCC-N				0.921

N0	761	380 (34.9)	381 (35.0)	
N1-3	543	268 (24.6)	275 (25.3)	
Others	874	441 (40.5)	433 (39.8)	
AJCC-M				0.938
M0	846	419 (38.5)	427 (39.2)	
M1	86	43 (3.9)	43 (3.9)	
Others	1246	627 (57.6)	619 (56.8)	

Footnotes: *MOR* (+) represented positive *MOR* mRNA expression; *MOR* (-) represented negative *MOR* mRNA expression; Young, under 60 years old; Old, over 60 years old; All the variables were described as frequencies and percentages, except for age described as mean and standard deviation.

Abbreviations: TCGA, the Cancer Genome Atlas; MOR, mu opioid receptor; AJCC, the American Joint Committee on Cancer; TNM, Tumor node metastasis.

Supplementary Table 3. Baseline characteristics of included patients by cancer type.

Characteristic	Type I<br>N = 2546	Type II<br>N = 2546	Type III<br>N = 1091	Type IV<br>N = 1091
MOR				
MOR (+)	366 (14.4)	444 (17.4)	175 (16.0)	127 (11.6)
MOR (-)	2180 (85.6)	2102 (82.6)	916 (84.0)	964 (88.4)
Age				
	60.2±13.7	57.8±15.0	58.6±14.8	59.4±12.7
Age group				
Young	1198 (47.1)	1342 (52.7)	546 (50.0)	544 (49.9)
Old	1318 (51.8)	1174 (46.1)	516 (47.3)	534 (48.9)
Unknown	30 (1.2)	30 (1.2)	29 (2.7)	13 (1.2)
Gender				
Female	1348 (52.9)	1162 (45.6)	545 (50.0)	549 (50.3)
Male	1192 (46.8)	1357 (53.3)	523 (47.9)	540 (49.5)
Unknown	6 (0.2)	27 (1.1)	23 (2.1)	2 (0.2)
Race				
Caucasian	1968 (77.3)	1743 (68.5)	807 (74.0)	704 (64.5)
African	215 (8.4)	242 (9.5)	103 (9.4)	126 (11.5)
Others	363 (14.3)	561 (22.0)	181 (16.6)	261 (23.9)
AJCC stage				
Stage I	808 (31.7)	422 (16.6)	273 (25.0)	252 (23.1)
Stage II	637 (25.0)	539 (21.2)	247 (22.6)	287 (26.3)
Stage III	516 (20.3)	389 (15.3)	182 (16.7)	194 (17.8)
Stage IV	282 (11.1)	189 (7.4)	158 (14.5)	60 (5.5)
Others	303 (11.9)	1007 (39.6)	231 (21.2)	298 (27.3)
Grade				
Low Grade	472 (18.5)	541 (21.2)	217 (19.9)	162 (14.8)
High Grade	494 (19.4)	597 (23.4)	194 (17.8)	205 (18.8)
Others	1580 (62.1)	1408 (55.3)	680 (62.3)	724 (66.4)
AJCC-T				
T1	713 (28.0)	390 (15.3)	208 (19.1)	245 (22.5)

T2	860 (33.8)	647 (25.4)	330 (30.2)	368 (33.7)
T3	597 (23.4)	657 (25.8)	253 (23.2)	310 (28.4)
T4	201 (7.9)	176 (6.9)	108 (9.9)	69 (6.3)
Others	175 (6.9)	676 (26.6)	192 (17.6)	99 (9.1)
AJCC-N				
N0	1243 (48.8)	1058 (41.6)	418 (38.3)	528 (48.4)
N1-3	886 (34.8)	570 (22.4)	342 (31.3)	325 (29.8)
Others	417 (16.4)	918 (36.1)	331 (30.3)	238 (21.8)
AJCC-M				
M0	1696 (66.6)	1050 (41.2)	531 (48.7)	595 (54.5)
M1	100 (3.9)	85 (3.3)	26 (2.4)	40 (3.7)
Others	750 (29.5)	1411 (55.4)	534 (48.9)	456 (41.8)

Footnotes: Young, under 60 years old; Old, over 60 years old; All the variables were described as frequencies and percentages, except for age described as mean and standard deviation.

Abbreviations: *MOR*, mu opioid receptor; AJCC, the American Joint Committee on Cancer; TNM, Tumor node metastasis.
